# Characterisation of X chromosome status of human extended pluripotent stem cells

**DOI:** 10.1111/cpr.13468

**Published:** 2023-05-17

**Authors:** Ying Wang, Ning Yang, Wen Sun, Chen Zhao, Xiaoxuan Hu, Shan Lu, Shiwei Cao, Nannan Wang, Tang Hai, Guihai Feng, Chenrui An, Haoyi Wang

**Affiliations:** ^1^ State Key Laboratory of Stem Cell and Reproductive Biology Institute of Zoology, Chinese Academy of Sciences Beijing China; ^2^ University of Chinese Academy of Sciences Beijing China; ^3^ Beijing Institute for Stem Cell and Regenerative Medicine Beijing China; ^4^ Institute for Stem Cell and Regeneration Chinese Academy of Sciences Beijing China; ^5^ Department of Obstetrics and Gynaecology Key Laboratory for Major Obstetric Diseases of Guangdong Province, Key Laboratory of Reproduction and Genetics of Guangdong Higher Education Institutes, The Third Affiliated Hospital of Guangzhou Medical University Guangzhou Guangdong China

## Abstract

Different pluripotent cell types have been established by capturing pluripotency in different states. Human extended pluripotent stem cells (hEPSCs), recently established by two independent studies, have the capability of differentiating into both embryonic and extraembryonic lineages, as well as forming human blastoids, showing great potential for early human development modeling and regenerative medicine. Considering that X chromosome status in female human pluripotent stem cells is dynamic and heterogeneous, and often leads to functional consequences, we characterized it in hEPSCs. We derived hEPSCs from primed human embryonic stem cells (hESCs) with defined X chromosome status (pre‐ or post‐X chromosome inactivation) using two previously published methods. We showed that hEPSCs derived using both methods had highly similar transcription profiles and X chromosome status. However, the X chromosome status of hEPSCs is largely determined by the primed hESCs from which they were derived, suggesting a lack of complete reprogramming of X chromosome during primed to extended/expanded pluripotency conversion. Furthermore, we found that the X chromosome status of hEPSCs affected their ability to differentiate into embryonic or extraembryonic lineage cells. Taken together, our work characterized the X chromosome status of hEPSCs, providing important information for the future application of hEPSCs.

## Graphical abstract

We found that the X chromosome status of human extended pluripotent stem cells (hEPSCs) were largely determined by the primed human embryonic stem cells (hESCs) from which they were derived, suggesting a lack of complete reprogramming of X chromosome during primed to extended/expanded pluripotency conversion. There were two distinct populations of hEPSCs derived from pre‐X chromosome inactivation (XCI) primed hESCs: HTG (with biallelic active X chromosomes and active RNA transcription) and LTG (with a partially monoallelic chromosome and prone to differentiation). hEPSCs derived from post‐XCI primed hESCs maintained monoallelic X chromosome status and lost XIST RNA coating. The chromosome status of hEPSCs affected their differentiation capability into embryonic or extraembryonic lineage cells.

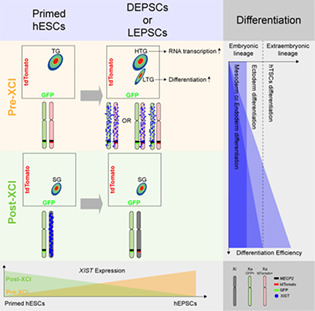

## 1 INTRODUCTION

During the early development of female mammals, epigenetic regulation of X chromosomes is very dynamic. This is particularly evident during the peri‐implantation period of human and mouse female embryos, where one of the two X chromosomes is randomly silenced via a process known as X chromosome inactivation (XCI).^1^ Abnormal XCI or misregulation of X‐linked gene expression often lead to foetal mortality,^2^, ^3^, ^4^ cancer,^5^, ^6^ autoimmunity,^7^ ageing,^8^ and heritable diseases.^9^ In female mice, paternally imprinted XCI occurs at the 8‐cell stage, and the silenced X is reactivated in the blastocyst epiblast, leading to two active X chromosomes in these cells. Random XCI then completes after implantation.^10^ Different from mice, in the ICM of human preimplantation embryos, both X chromosomes remain active with a biallelic expression of *XIST* and reduced expression of X‐linked genes.^11^, ^12^ Due to the lack of a suitable model, the process and mechanism of X chromosome regulation during early human development remain elusive.

Pluripotent stem cells (PSCs) have become important model to study early embryonic development since they can differentiate into all cell lineages of the body. Different pluripotent cell types have been established by capturing pluripotency in different states.^13^, ^14^, ^15^, ^16^ Conventional human embryonic stem cells (hESCs) exhibit primed pluripotency that resembles post‐implantation epiblast. Regarding the X chromosome status, primed hESCs demonstrate notable heterogeneity among different cell lines,^17^ and are classified into three classes.^18^, ^19^ Class I hESCs have two active X chromosomes with no *XIST* expression (Xa^XIST−^Xa^XIST−^), which can be maintained in the hypoxic environment.^20^, ^21^, ^22^ The other two classes of hESCs have undergone XCI. In Class II hESCs, the inactivated X chromosome is coated by XIST (Xa^XIST−^Xi^XIST+^). With long‐term in vitro culture, hESCs gradually lose *XIST* expression with partial erosion of previously silenced X‐linked genes, classified as Class III (Xa^XIST−^Xe^XIST−^).^23^, ^24^ Given the heterogeneity of X chromosome status, primed hESC is not an ideal model for studying human XCI.

Human naive pluripotency, representing the pre‐implantation epiblast, has been established using small molecular compound combinations or transgene overexpression,^25^, ^26^, ^27^, ^28^, ^29^, ^30^, ^31^, ^32^, ^33^ among which, the naive hESCs derived using 5iLA^34^, ^35^ and t2iLGö^33^ systems are more similar to human preimplantation epiblast.^36^, ^37^, ^38^, ^39^ Previous studies found that naive hESCs have two active X chromosomes, but exhibit heterogeneous expression patterns of *XIST* and non‐random XCI after differentiation.^36^, ^37^, ^38^ By completely blocking FGF signalling, homogenous naive hESCs are derived, which biallelically express *XIST* and initiate random XCI upon differentiation.^39^

Lately, a new type of pluripotent human PSCs was established by two groups independently through small‐molecule compound screening and signalling pathway analysis.^40^, ^41^ In addition to embryonic tissue lineage, these cells can also differentiate into extraembryonic lineage, therefore are termed human extended (or expanded) pluripotent stem cells (hEPSCs).^40^, ^41^ Previous studies found that hEPSCs increased the efficiency of chimera formation^40^, ^42^ and were capable of forming synthetic human blastocyst‐like structures,^43^ representing a promising model for studying early human development. However, the X chromosome status of hEPSCs has not been well characterized, hindering their applications in developmental biology and regenerative medicine.

Here, we characterized the X chromosome status of hEPSCs derived from primed hESC lines with defined X chromosome status (pre‐ or post‐XCI), using both previously published hEPSC culture systems. Our findings show that the X chromosome status of hEPSCs cultured in both culture systems is highly similar. However, the X chromosome status of hEPSCs resembles that of the primed hESCs from which they were derived, and affects the differentiation efficiency towards trophoblasts and ectoderm lineage.

## 2 MATERIALS AND METHODS

### 2.1 Culture of naive hESCs

WIBR3^MGT^ HT naive hESCs were established in our laboratory.^39^ This feeder‐dependent naive hESCs lines were cultured in 5iLA culture medium (N2B27 medium and supplemented with 5iLA) under 5% O_2_, 5% CO_2_ at 37°C. Naive hESCs were maintained and expanded as described previously.

### 2.2 Culture of primed hESCs

To convert naive hESCs into primed hESCs, HT naive hESCs were isolated into single cells (5 × 10^5^) on a feeder layer in Essential 8 medium (E8) and supplemented with Y‐27632 (10 μM). On the second day, Y‐27632 was withdrawn. After 5 days, primed‐like clones were observed and were passaged as clusters onto pre‐coated Matrigel for subsequent culturing. TG, SG primed hESCs were sorted by flow cytometry for the differences of their reporter gene expression and identified by co‐staining of RNA fluorescence in situ hybridization (FISH) and Immunocytochemistry. For stable passages, Class I TG and Class II SG primed hESCs were maintained at 37°C under 5% O_2_, 5% CO_2_, and 20% O_2_, 5% CO_2_, respectively.

### 2.3 Culture of hEPSCs

Currently, two laboratories have established hEPSCs in distinct culture systems.^40^, ^41^ To distinguish these two culture systems, DEPSCs and LEPSCs were used to represent them. To obtain hEPSCs, primed hESCs (5 × 10^5^) were seeded on the feeder layer in E8 and supplemented with Y‐27632(10 μM). On the second day, the medium was switched to N2B27‐LCDM medium^40^ and CHAX^41^ prepared as described previously. DEPSCs and LEPSCs were passaged every 3 days using Accutase.

### 2.4 Flow cytometry analysis

Primed hESCs (Class I TG and Class II SG primed hESCs) and their hEPSCs (DEPSCs and LEPSCs) were separated into a single cell suspension on LSR II SORP (Beckton–Dickinson) for population analysis. tdTomato was excited by a Coherent Compass 561 nm (25 mW) yellow/green laser, using a bandpass filter of 610/20. Green fluorescent protein (GFP) was excited by a Coherent Sapphire Solid State 488 nm (100 mW) blue laser, using a bandpass filter of 525/50. Data analysis were performed using FlowJo (version 10.6.2).

### 2.5 RNA‐FISH and immunofluorescence (IF) co‐staining

Cells were cultured on slides. *XIST* probe and *ATRX* probe were respectively generated from *XIST* exon 1 DNA (GenBank U80460: 61251–69,449) and BACs including *ATRX* (RP11‐42 M11, BACPAC), which were labelled using nick translation kit (Roche) with Cy3‐dUTP (Amersham) and Cy5‐dUTP (Exon bio), respectively. RNA‐FISH was carried out as described previously.^38^ We detected H3K27me3 (FITC 488) enrichment in cells with IF assays. Nuclear DNA was labelled by DAPI. Finally, slides were visualized with Leica TCS Sp8 confocal microscope (Zeiss) equipped with filters that are compatible for imaging with DAPI, Cy3, and Cy5.

### 2.6 IF staining

To perform immunocytochemical analysis, cells were fixed with 4%PFA (pH = 7.4) for 30 min at room temperature. Fixed cells were washed three times with PBS and were incubated in 1% TritonX‐100 for 30 min, followed by blocking with 2% BSA for 1 h. Then, immunostaining was performed according to standard protocols using the following primary antibodies: OCT3/4, NANOG, KLF4, SP5, GATA3, KRT7, Brachyury, PAX6, and SOX17. Appropriate Alexa Fluor dye‐conjugated secondary antibodies (Invitrogen) were used. Nuclei were stained with DAPI (Life Technologies). Images were taken with a confocal microscope (Zeiss). Data analysis were performed with ImageJ.

### 2.7 Alkaline phosphatase (AP) staining

AP activity in hPSCs was detected by a BCIP/NBT kit (BOSTER) according to the manufacturer's instructions. Briefly, the cells were washed twice with PBS, fixed with 4% PFA/PBS (pH 7.4) for 10 min at room temperature, and washed three times with PBS. Then, the cells were incubated with a mixture (1 mL H_2_O/one drop of A/one drop of B) for 30 min at room temperature. The AP‐positive colonies showed a dark violet colour and were photographed with a Nikon inverted microscope.

### 2.8 RNA‐seq library preparation and data analysis

To prepare RNA for sequencing, one million primed hESCs or hEPSCs were trypsinized. Total RNA was isolated using Trizol. Sequencing was performed on an Illumina X Ten sequencer with a 150 bp paired‐end sequencing reaction. And the RNA sequencing data of references were downloaded from ArrayExpress. All the RNA‐seq data were aligned to the human reference genome assembly (hg38), using STAR^44^ (version 2.7.1a) with default parameters, and a customized script was used to filter the uniquely mapped reads. Normalized gene expression level (fragments per kilobase million or FPKM) was obtained using Stringtie^45^ (version 2.0), and differentially expressed gene (DEG) analysis was performed using DESeq2^46^ with default parameters, two‐fold changes of gene FPKM and *p*‐value <0.05 were used as the cutoff values. Genes with no <1 FPKM in at least one sample were used for the following analysis. Gene FPKM values were transformed by log2 and used to produce scatterplots by R. The R 'sva' function was used to eliminate the batch effect, 'scatterplot3d' and 'prcomp' functions were used to show the principal component analysis (PCA) results. Heatmap analysis was performed with 'pheatmap' function in R. Gene‐enrichment and functional annotation analysis were performed using the David tool.^47^ In order to analyse allele‐specific expression of X‐linked genes, we use 'pysamstats' (https://github.com/alimanfoo/pysamstats) to count the reads covering X‐linked single nucleotide polymorphisms (SNPs). The frequency of occurrence of the reference nucleotide was then calculated to distinguish whether the X‐linked genes involved were biallelically or monoallelically expressed. The calculation formula is: frequency = reads covering reference nucleotide/(reads covering reference nucleotide + reads covering alterative nucleotide). Transcripts expressing <1 FPKM were classified as 'not expressed'. Transcripts were classified as biallelic when the minor allele frequency was between 25% and 75%. Transcripts were classified as monoallelic when the minor allele frequency was <25% The *X*:*A* ratio of samples was calculated using the sum of gene expression on ChrX versus the sum of gene expression on Chr1. Gene Set Enrichment Analysis (GSEA) was performed with GSEA software (version 4.0.3).^48^

### 2.9 Characterisation of X‐linked SNPs expression

WIBR3 data interrogated by Affymetrix human SNPs array 6.0 were used.^20^ Array intensity data were analysed by Affymetrix Genotyping Console. Total RNA was isolated and synthesized cDNA with reverse transcription. Three SNPs were used to characterize their distribution on the X chromosome. About 500 bp fragments encompassing SNPs sites were amplified by primers (Table S1) using Taq DNA polymerase. The PCR products were sequenced. The sequence analysis was performed with SnapGene (version 5.0.5).

### 2.10 In vitro differentiation assay

For human trophoblast stem cells (hTSCs) differentiation, hEPSCs(DEPSCs and LEPSCs) derived from different X state hESCs were isolated into single cells as described above; 0.2 × 10^4^ well^−1^ were plated on collagen IV‐coated culture plated in hTSCs medium as described.^49^ After 5 days, hTSCs‐like clonies were observed. After four passages, the cells were stained with antibodies recognising specific TSCs markers, and then analysed for differentiated efficiency using HCA software. The differentiation efficiency was determined based on the ratio of cells expressing specific marker versus total nucleated cells.

For differentiation towards three germ layers, single hEPSCs (DEPSCs and LEPSCs) with different X states were plated on Matrigel‐coated plates at a density of 2 × 10^4^ well^−1^ in three germ layer cells medium for 7 days culture.^50^ Then, the differentiated cells were stained with antibodies recognising specific lineage markers, and then analysed for differentiated efficiency using HCA software.

For fibroblast differentiation, we used standard protocol published previously.^34^ In brief, hEPSCs(DEPSCs/LEPSCs) derived from different X state hESCs were first re‐primed, respectively. Then, these re‐primed hESCs were digested into clumps with EDTA and plated onto gelatin pre‐coated using fibroblast culture medium supplemented with Y‐27632 (10 μM) for 5 days culture. The fluorescent‐reporter changes were detected by fluorescence activated cell sorting (FACS).

### 2.11 Reverse‐transcription polymerase chain reaction (RT‐PCR)

Total RNA was purified using Trizol and complementary DNA (cDNA) was generated using SuperScript III First‐Strand Synthesis SuperMix kit and 1 μg of total RNA according to the manufacturing protocols. RT‐PCR analysis of specific gene expression in hEPSCs‐ (*SP5*, *DMD*, and *WLS*) and hTSCs‐specific markers (*GATA3*, *KRT7*, *CDX2*, *ELF5*, *TP63*, and *TFAP2C*), and endoderm markers (*AFP*, *FOXA2*, and *SOX17*), mesoderm markers (*Mixl1*, *Brachyury*, and *MESP1*) and ectoderm markers (*PAX6*, *Notch1*, and *SOX1*) were performed using gene‐specific primers (Table S2) and SYBR Green PCR Master Mix in ABI Primsm7300 (Bio‐Rad). Results were normalized to *GAPDH* transcripts and analysed using 2^−ΔΔ𝐶𝑡^ method.

### 2.12 Quantification and statistical analysis

FACS data analysis were performed using FlowJo. Prism Graph Pad 9.0 was used to perform statistical analysis and data plotting. For the quantification of RNA‐FISH and H3K27me3 IF staining, we randomly selected 100 nuclei for signal collection. Differentiation assay was performed by HCA software. All experiments were performed with no less than two biological replicates. Statistical analyses were performed with a two‐tailed Student's *t*‐test and *p‐*value <0.05 was considered significant. *, **, ***, **** denote significance at 0.05, 0.01, 0.001, 0.0001 levels, respectively.

## 3 RESULTS

### 3.1 Characterisation of pre‐ and post‐XCI primed hESCs carrying dual X reporters

**FIGURE 1:**
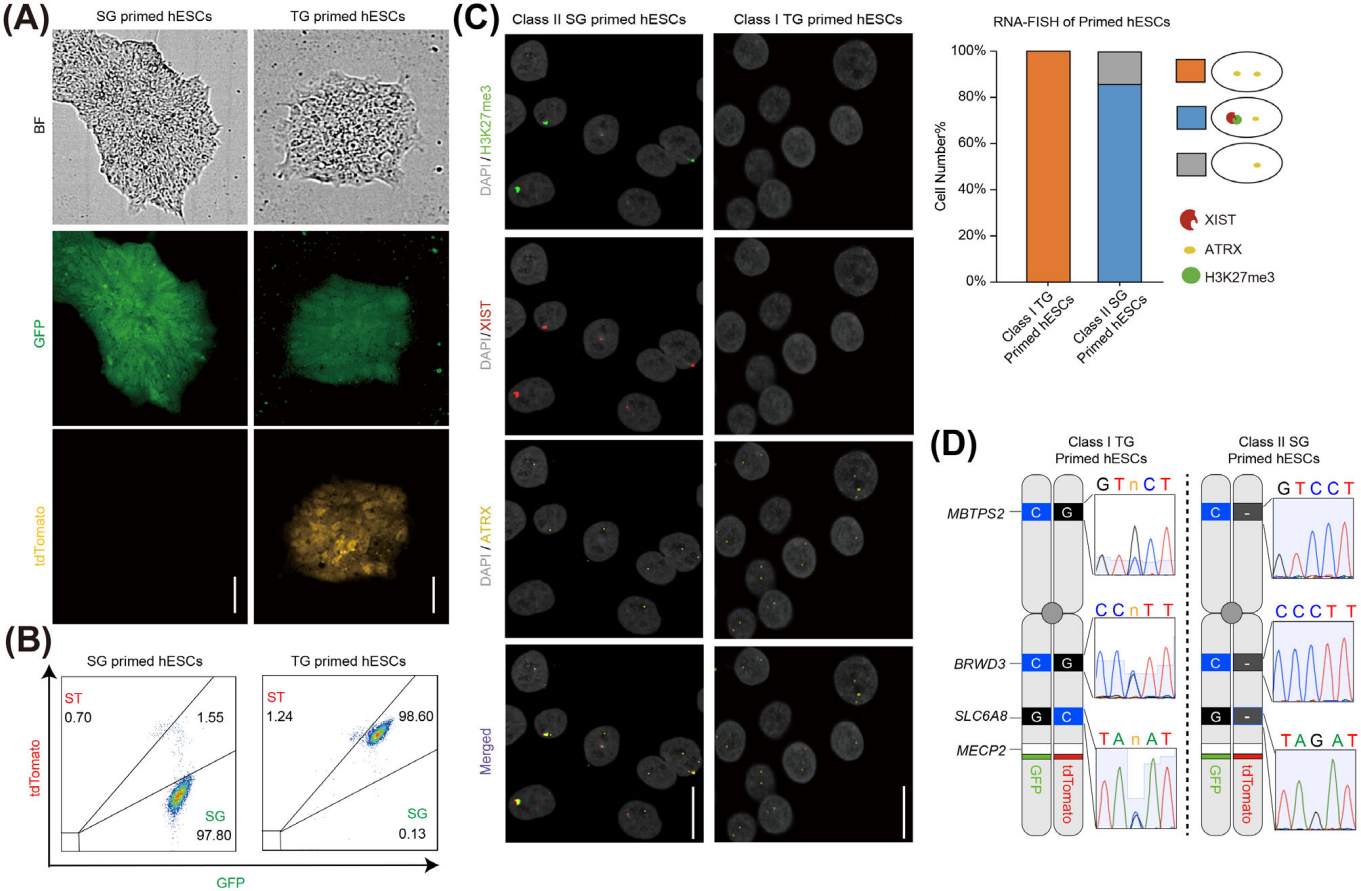
Characterisation of pre‐ or post‐XCI primed hESCs. (A) Representative images of WIBR3^MGT^ TG and SG primed hESCs. Scale bars indicate 100 μm. (B) The GFP and tdTomato expression levels of WIBR3^MGT^ TG and SG hESCs by flow cytometric analysis. (C) Left panel: representative RNA‐FISH images of WIBR3^MGT^ TG and SG hESCs, detecting XIST (Red) and ATRX (Yellow) and H3K27me3 (Green) by IF co‐staining. Scale Bars indicate 10 μm. Right panel: quantification of cells having different RNA‐FISH and IF co‐staining patterns (depicted by the cartoon on the right). (D) Position of 3 predefined X‐linked SNPs in WIBR3. Different colours represent different bases. Sequencing peaks of 3 random SNPs in cDNA extracted from WIBR3^MGT^ TG and SG hESCs. GFP, green fluorescent protein; hESCs, human embryonic stem cells; IF, immunofluorescence; XCI, X chromosome inactivation.

In this study, we aimed to derive hEPSCs from primed hESCs line WIBR3^MGT^ carrying dual X reporters (GFP and tdTomato).^35^, ^39^ Given that the X chromosome status is dynamic and heterogeneous in different pluripotent states, we hypothesize that the initial X chromosome status of the cells from which hEPSCs are derived could have an effect. Therefore, we first generated primed hESCs lines with defined X chromosome status. As described in our previous work^39^ (Figure 1A), we differentiated HT naive hESCs into pre‐XCI (tdTomato and GFP double positive, TG) and post‐XCI (single GFP positive, SG) primed hESCs lines (Figure 1B). We examined the X chromosome status in both cell lines by co‐staining nascent transcripts of *XIST* and *ATRX* (a non‐escaping X‐linked gene) using RNA FISH‐ along with IF staining of histone modification H3K27me3. Two ATRX transcription spots per nucleus were detected in all the TG primed hESCs without any XIST cloud or H3K27me3 focus (Figure 1C), suggesting that both Xs remained active and these cells were in the Xa^XIST‐^Xa^XIST‐^ state, classified as Class I primed hESCs. On the other hand, the majority of SG primed hESCs showed a single XIST focus co‐localized with H3K27me3 signal and one separate ATRX focus per nucleus (Figure 1C). This implied that the cells had initiated XCI and resided in the Xa^XIST‐^Xi^XIST+^ state, classified as Class II primed hESCs.

To further examine the allele‐specific expression of X‐linked genes, we sequenced the transcripts containing three X‐linked SNPs in WIBR3,^20^ and found biallelic expression in TG primed hESCs and monoallelic expression on the GFP‐targeted X chromosome in SG primed hESCs in all three cases (Figure 1D). These results confirm that TG primed hESCs are pre‐XCI (Class I), and SG primed hESCs are post‐XCI (Class II) with the tdTomato‐targeted X chromosome being silenced.

### 3.2 Characterisation of X chromosome status of hEPSCs

Previously, two hEPSC derivation methods were established by Deng group^40^ and Liu group,^41^ and we named them DEPSCs and LEPSCs, respectively, in this study. These two systems were used to derive hEPSCs from WIBR3^MGT^ Class I TG or Class II SG primed hESCs, and the X‐linked reporter expression in hEPSCs at different passages during induction was characterized. The expression pattern of X‐linked reporters and their X status began to stabilize at P5 and was maintained through P10 (Figure S1A,B). We successfully derived four hEPSCs lines after ten passages, which we termed CI‐DEPSCs/LEPSCs and CII‐DEPSCs/LEPSCs. These EPSCs had high AP activity (Figure S1C) and similar expression levels of pluripotent genes (Figure S1D), indicating that these hEPSCs maintained pluripotency.

By comparing published RNA‐seq datasets from DEPSCs,^40^ LEPSCs,^41^ H1 primed hESCs and HT 5iLA naive hESCs,^39^ we found that 236 genes were specifically upregulated in hEPSCs (Figure S1E,F). Gene ontology (GO) analysis showed these genes were enriched in multiple biological processes involved in RNA metabolic and synthesis processes (Figure S1G), suggesting that hEPSCs have more active RNA transcription than naive or primed hESCs. Higher expression levels of hEPSC‐specific genes (*SP5*, *DMD* and *WLS*) were identified in CI‐ and CII‐hEPSCs than in primed or naive hESCs using RT‐PCR and IF staining (Figure S1H,I), confirming the successful establishment of hEPSCs. Furthermore, we found that DEPSCs and LEPSCs expressed hEPSCs‐specific genes at similar levels (Figure S1H), regardless of the culture systems or the X chromosome status.

**FIGURE 2:**
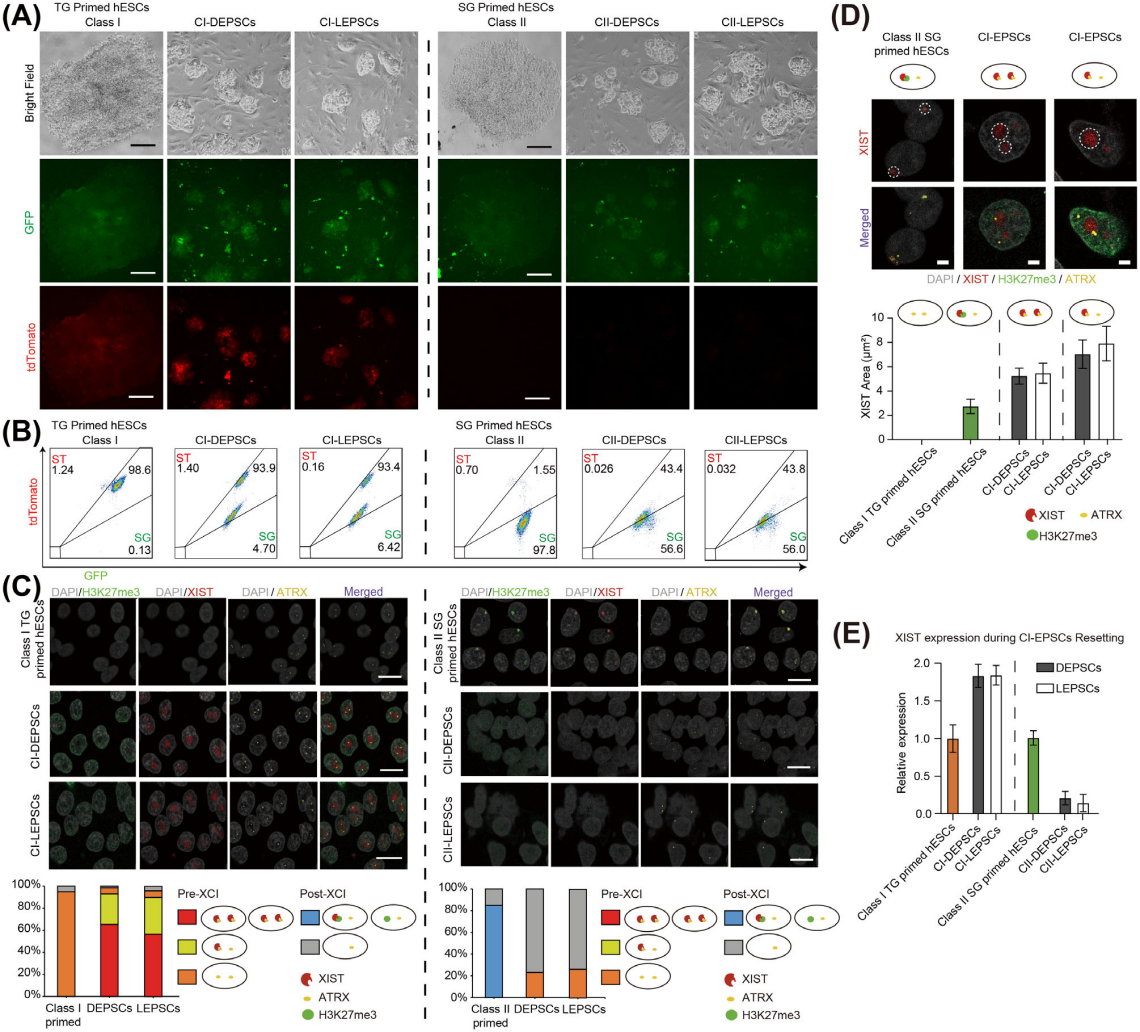
Characterisation of X status of hEPSCs derived from pre‐ or post‐XCI primed hESCs. A. Representative images of Class I TG, Class II SG primed hESCs and their DEPSCs or LEPSCs derivatives at P10. Scale bars indicate 100 μm. B. FACS analysis showing the GFP and tdTomato activities in DEPSCs and LEPSCs derived from Class I TG or Class II SG primed hESCs. C. Upper panel: representative RNA‐FISH and IF co‐staining images of Class I TG, Class II SG primed hESCs and their DEPSCs or LEPSCs derivatives to detect XIST (Red), ATRX (Yellow) and H3K27me3 (Green). Scale bars indicate 10 μm; Lower panel: quantification analysis of cells having different patterns with XIST, ATRX and H3K27me3 by RNA‐FISH and IF co‐staining. D. Upper panel: representative RNA‐FISH images of cells expressing *XIST*. Scale bars indicate 2 μm. Lower panel: quantification of XIST transcript territories area (μm^2^) in CI‐hEPSCs and Class II SG primed hESCs. The XIST area could not be quantified in Class I TG primed hESCs. E. RT‐PCR analysis of *XIST* expression levels of Class I TG，Class II SG primed hESCs and their descendants hEPSCs. FACS, fluorescence activated cell sorting; GFP, green fluorescent protein; hESCs, human embryonic stem cells; IF, immunofluorescence; XCI, X chromosome inactivation.

Based on the X‐linked reporter expression, CI‐DEPSCs and CI‐LEPSCs were both double positive, while CII‐DEPSCs and CII‐LEPSCs were largely single GFP positive with a slight elevation of tdTomato signal (Figure 2A,B), suggesting that hEPSCs derived from the same primed hESCs shared similar X chromosome status. Although CI‐hEPSCs were double positive, two distinct populations of cells were present: one with low X reporter expression (LTG) and the other with higher X reporter expression (HTG) (Figure 2B). We sorted these two populations and found that LTG cells could not be stably maintained in hEPSCs medium. On the other hand, the sorted HTG cells could be further cultured, but gradually turned into two populations of HTG and LTG cells again (Figure S1J), indicating that neither DEPSCs nor LEPSCs mediums could suppress this heterogeneity. In contrast, CII‐hEPSCs derived from post‐XCI primed hESCs showed one population of single GFP positive cells with slightly higher tdTomato expression than Class II SG primed hESCs (Figure 2B), suggesting that X chromosomes were not completely reactivated during the primed hESCs to hEPSCs transition.

To further characterize the X chromosome status of hEPSCs, we performed RNA‐FISH of *XIST*, *ATRX*, as well as IF staining of H3K27me3. Consistently, similar expression patterns of *XIST* or *ATRX* and H3K27me3 modification were detected between DEPSCs and LEPSCs derived from the same class primed hESCs (Figure 2C), indicating no differences in X chromosome status between DEPSCs and LEPSCs. However, hEPSCs derived from Class I or II primed hESCs exhibited notable differences. In CI‐hEPSCs, over 90% of cells had two ATRX foci and the majority of the cells had one or two XIST clouds without any H3K27me3 focus (Figure 2C), suggesting that the two X chromosomes remained active and gained *XIST* expression, resulting in Xa^XIST+^Xa^XIST+^ or Xa^XIST+^Xa^XIST−^ states. In CII‐hEPSCs, ~80% of cells had a single ATRX focus per nucleus (Figure 2C), indicating a single active X chromosome in these cells. However, the rest of the cells had two ATRX foci, presumably representing the cells that slightly upregulated tdTomato expression. Notably, XIST or H3K27me3 foci in CII‐hEPSCs were not detected in CII‐hEPSCs (Figure 2C). These results demonstrate that most CII‐hEPSCs cannot completely reactivate the silenced X chromosome and lose the coating of *XIST* and H3K27me3 modification on it.

Based on the RNA‐FISH results, we calculated the area of the XIST cloud in CI‐hEPSCs, and found that the XIST clouds in CI‐hEPSCs were more diffused than in Class II post‐XCI primed hESCs (Figure 2D), which was similar to that in HT naive hESCs.^39^ We further quantified the *XIST* expression of CI‐ or CII‐hEPSCs using RT‐PCR (Figure 2E). Consistent with the RNA‐FISH results, the *XIST* expression levels increased in CI‐hEPSCs, while downregulated in CII‐hEPSCs, compared to primed hESCs (Figure 2E).

Collectively, these results demonstrate that despite of the different derivation methods, DEPSCs and LEPSCs exhibit the similar X chromosome status, and hEPSCs derived from pre‐ or post‐XCI primed hESCs execute different *XIST* expression regulation: CI‐hEPSCs gain *XIST* expression, while CII‐hEPSCs lose *XIST* expression.

### 3.3 Transcriptome analysis of hEPSCs

**FIGURE 3:**
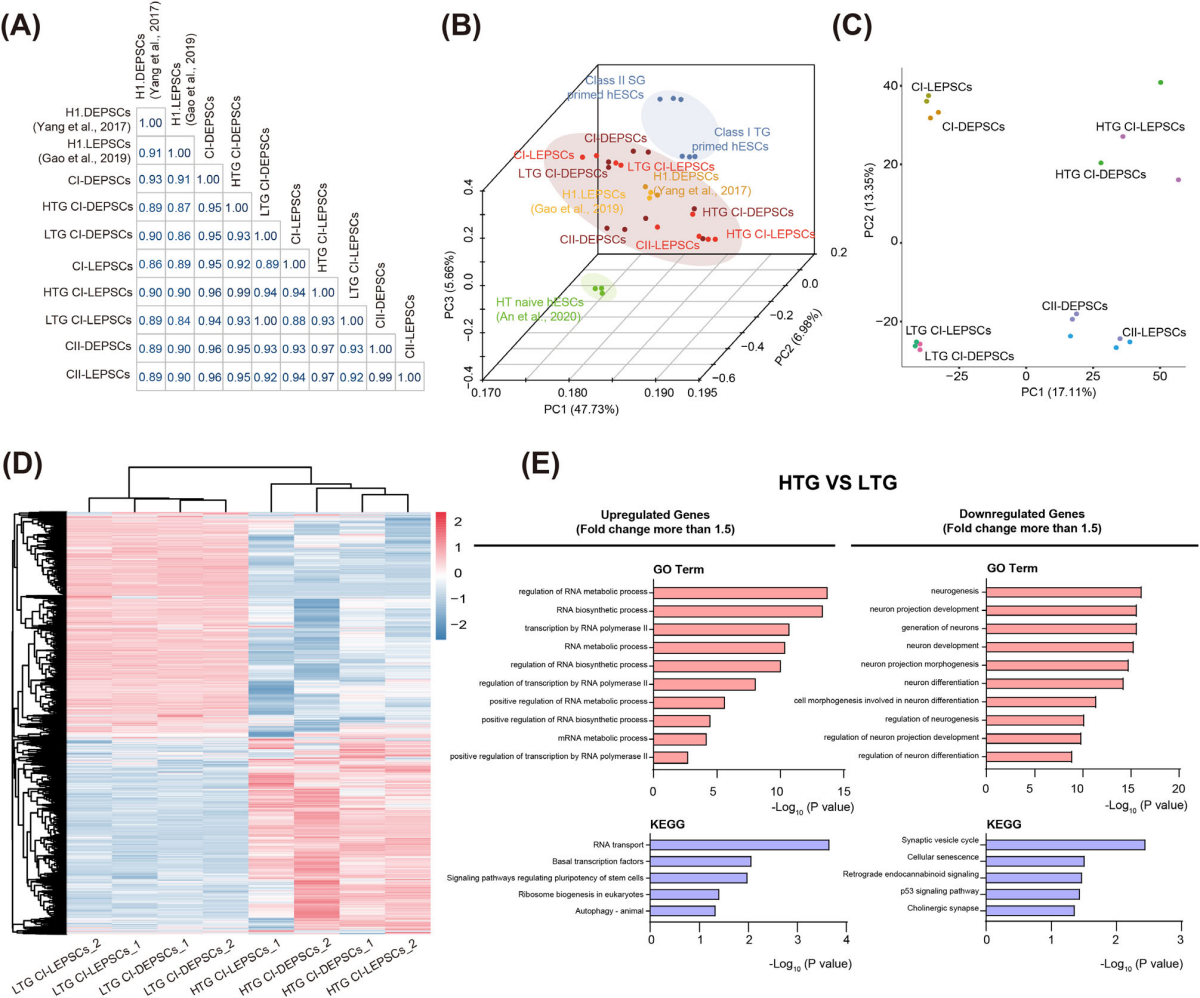
Comparison of the transcriptome of hEPSCs derived from Class I TG or Class II SG primed hESCs. (A) Correlation plot of gene expression of hEPSCs (DEPSCs, LEPSCs) derived from WIBR3^MGT^ Class I TG, Class II SG primed hESCs and H1 hEPSCs.^40^, ^41^ Spearman's test was used to calculate their correlation level. (B). 3D PCA of RNA‐Seq data from hEPSCs comparing with human primed and HT naive hESCs.^39^ Every single dot represents one sample in each cell line. (C) PCA of RNA‐Seq data from hEPSCs. (D) Heatmap of DEGs expression between WIBR3^MGT^ HTG and LTG CI‐hEPSCs. The DEGs were selected with FPKM fold change more than 1.5 between two types of cells. (E) Using the selected DEGs between HTG and LTG CI‐hEPSCs, GO term and KEGG analysis were both performed by clusterProfiler in R. X‐axis represents the enrichment fold of genes. Y‐axis represents either the biological processes or signalling pathways for the enriched genes. DEG, differentially expressed gene; FPKM, fragments per kilobase million; GO, gene ontology; hEPSCs, human extended pluripotent stem cells; hESCs, human embryonic stem cells; HTG, higher X reporter expression; KEGG, Kyoto Encyclopedia of Genes and Genomes; LTG, low X reporter expression; PCA, principal component analysis.

We performed RNA‐seq on HTG and LTG CI‐hEPSCs, as well as CII‐hEPSCs. Compared to the published datasets from H1 DEPSCs^40^ and H1 LEPSCs,^41^ we found that all hEPSCs samples shared a high degree of correlation (Figure 3A), while hEPSCs were consistently more similar to primed hESCs than to HT naive hESCs at transcriptome levels (Figure S2A). PCA nicely separated each cell type and showed that the HT naive hESCs^39^ differed the most from the Class I TG or Class II SG primed hESCs (Figure 3B). Lying in between naive and primed pluripotency, all hEPSCs, including CI‐DEPSCs/LEPSCs, HTG CI‐DEPSCs/LEPSCs, LTG CI‐DEPSCs/LEPSCs, CII‐DEPSCs/LEPSCs, and H1 DEPSCs/LEPSCs were positioned closely together (Figure 3B).

Next, we analysed the DEGs between CI‐ and CII‐hEPSCs. Clustering analysis showed the similarity of gene expression between DEPSCs and LEPSCs derived from the same cells, while separating the hEPSCs derived from Class I or II primed hESCs using these DEGs (Figure S2B). We further performed GO term and Kyoto Encyclopedia of Genes and Genomes (KEGG) pathway analyses of the DEGs. The upregulated genes of CI‐ or CII‐hEPSCs were consistently enriched in similar biological processes and signalling pathways (Figure S2C and S2D).

Since these hEPSCs lines were derived from primed hESCs with different X chromosome statuses, using two culture systems, we performed PCA among all of the hEPSCs to identify the key factors differentiating them. We found that hEPSCs derived from the same primed hESCs were placed together (CI‐DEPSCs to CI‐LEPSCs and CII‐DEPSCs to CII‐LEPSCs) (Figure 3C), indicating that the two hEPSCs culture systems generate highly similar cell lines. However, the X chromosome status of the initiating primed hESCs has a major effect on the transcriptional profiling of the hEPSCs. Furthermore, the HTG CI‐hEPSCs clearly differed from the LTG CI‐hEPSCs, while un‐sorted CI‐hEPSCs were lying between these two cell populations (Figure 3C), demonstrating the heterogeneity of the CI‐hEPSCs. Differential gene analysis showed that 2816 genes were unpregulated in HTG CI‐hEPSCs, and 3135 genes were upregulated in LTG CI‐hEPSCs (Figure 3D). We next characterized the DEGs between HTG and LTG CI‐hEPSCs, and performed the GO term and KEGG analysis. The genes upregulated in HTG CI‐hEPSCs were enriched in RNA metabolic and synthesis processes (Figure 3E), many of which were upregulated specifically in hEPSCs (Figure S1G). In contrast, the genes upregulated in LTG CI‐hEPSCs were enriched in neurogenesis‐related ontology (Figure 3E), indicating that LTG CI‐hEPSCs are prone to neuronal differentiation and therefore could not be long‐term maintained after cell sorting.

### 3.4 The X‐linked gene expression of hEPSCs is affected by the original X chromosome status of primed hESCs

**FIGURE 4:**
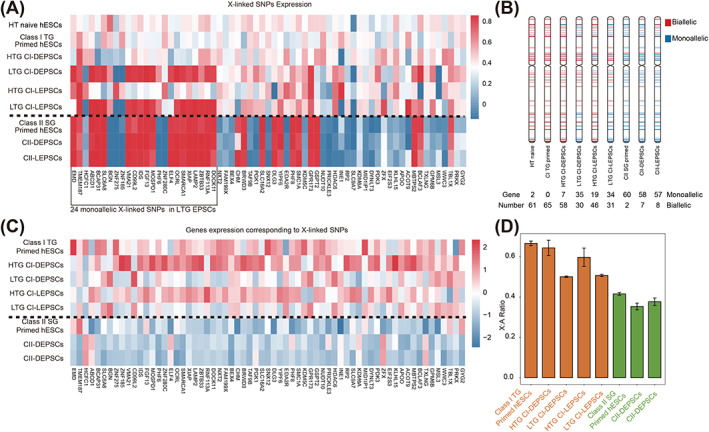
Detection of X‐linked gene expression in hEPSCs. (A) Heatmap of allelic expression of X‐linked genes in HT naive hESCs.^39^ Class I TG and Class II SG primed hESCs and their generating hEPSCs, based on SNPs reads covering X‐linked genes. (B) Schematic of X chromosomes that summarizes the results from allelic analysis of SNPs reads for HT naive hESCs.^39^ hEPSCs derived from Class I TG, Class II SG primed hESCs, respectively. Monoallelic (blue, <25% from minor allele), biallelic (red, 25%–75% from minor allele), or not expressed (grey, <1 FPKM/Sample). The number of monoallelic and monoallelic genes is shown below. (C) Heatmap showing the expression of X‐linked genes referring to SNPs analysis. (D) *X*:*A* ratio of gene dosage expression shown in histogram. Every column represents the ratio of FPKM_ChrX gene_ to FPKM_Chr1 gene_. FPKM, fragments per kilobase million; hEPSCs, human extended pluripotent stem cells; hESCs, human embryonic stem cells; SNP, single nucleotide polymorphisms;

To further investigate the X‐linked gene expression of hEPSCs, we performed a SNPs analysis across the entire X chromosome. For hEPSCs lines derived from Class I primed hESCs, HTG CI‐hEPSCs showed a similar level of biallelic expression as the HT naive or Class I TG primed hESCs (Figure 4A), indicating that both X chromosomes remained active, consistent with our RNA‐FISH data (Figure 2C). In comparison, only a portion of the X‐linked SNPs was biallelically expressed in LTG CI‐hEPSCs, with 24 X‐linked SNPs remaining monoallelic (Figure 4A). GO term analysis revealed these 24 genes were associated with RNA transcription (Figure S3A). Considering that LTG and HTG CI‐hEPSCs were both derived from Class I TG primed hESCs which maintain two active Xs, these results suggest that partial XCI was initiated in LTG CI‐hEPSCs, perhaps due to their propensity to differentiate (Figure 3E).

CII‐hEPSCs and Class II SG primed hESCs exhibited strict monoallelic expression across the entire X chromosome (Figure 4A), indicating the maintenance of post‐XCI status, and the absence of reactivation of silenced X chromosome during hEPSC derivation. This conclusion was further supported by the calculation of the number of X‐linked genes expressed mono‐ or biallelically (Figure 4B). Interestingly, the monoallelic genes in LTG CI‐hEPSCs were predominantly located at the end of the long arm of the X chromosome (Figure 4B), suggesting that these genes were preferentially silenced.

Furthermore, we quantified the expression of X‐linked genes containing SNPs. There was no significant difference between HTG CI‐hEPSCs and Class I TG primed hESCs, both of which carry two active X chromosomes (Figure 4C). Compared to HTG CI‐hEPSCs, LTG CI‐hEPSCs showed similar expression levels of genes expressed biallelically, while lower levels of genes corresponding to monoallelic SNPs (Figure 4C), indicating partial silencing and downregulation of these 24 genes in LTG CI‐hEPSCs. As expected, almost all X‐linked gene expression was at a lower level in CII‐hEPSCs or Class II SG primed hESCs, compared to CI‐EPSC or Class I TG primed hESCs (Figure 4C), due to their post‐XCI status.

In addition, we calculated the ratio of X‐linked gene expression to autosome genes expression (X:A ratio). X:Aratios of the CII‐hEPSCs or Class II SG primed hESCs were closed to 0.4, while HTG CI‐hEPSCs and Class I primed hESCs had a higher ratios above 0.6 (Figure 4D). Owing to the partial XCI, LTG CI‐hEPSCs was lower than that of HTG CI‐hEPSCs (Figure 4D).

These results demonstrate that significant variation in X‐linked gene expression among different hEPSCs lines, and they largely maintained the X chromosome status of the primed hESCs from which they were derived.

### 3.5 hEPSCs with different X chromosome statuses have different differentiation capabilities

To investigate whether hEPSCs undergo random XCI, we differentiated them into fibroblasts and examined the expression of X‐linked reporters. We found that most fibroblasts derived from CI‐hEPSCs remained double positive, whereas fibroblasts differentiated from CII‐hEPSCs maintained GFP expression and weak expression of tdTomato, similar to CII‐hEPSCs (Figure S4A). These results suggest that hEPSCs do not undergo random XCI after differentiation in vitro, and that the *X* chromosomes of the differentiated cells maintained a status similar to hEPSCs.

**FIGURE 5:**
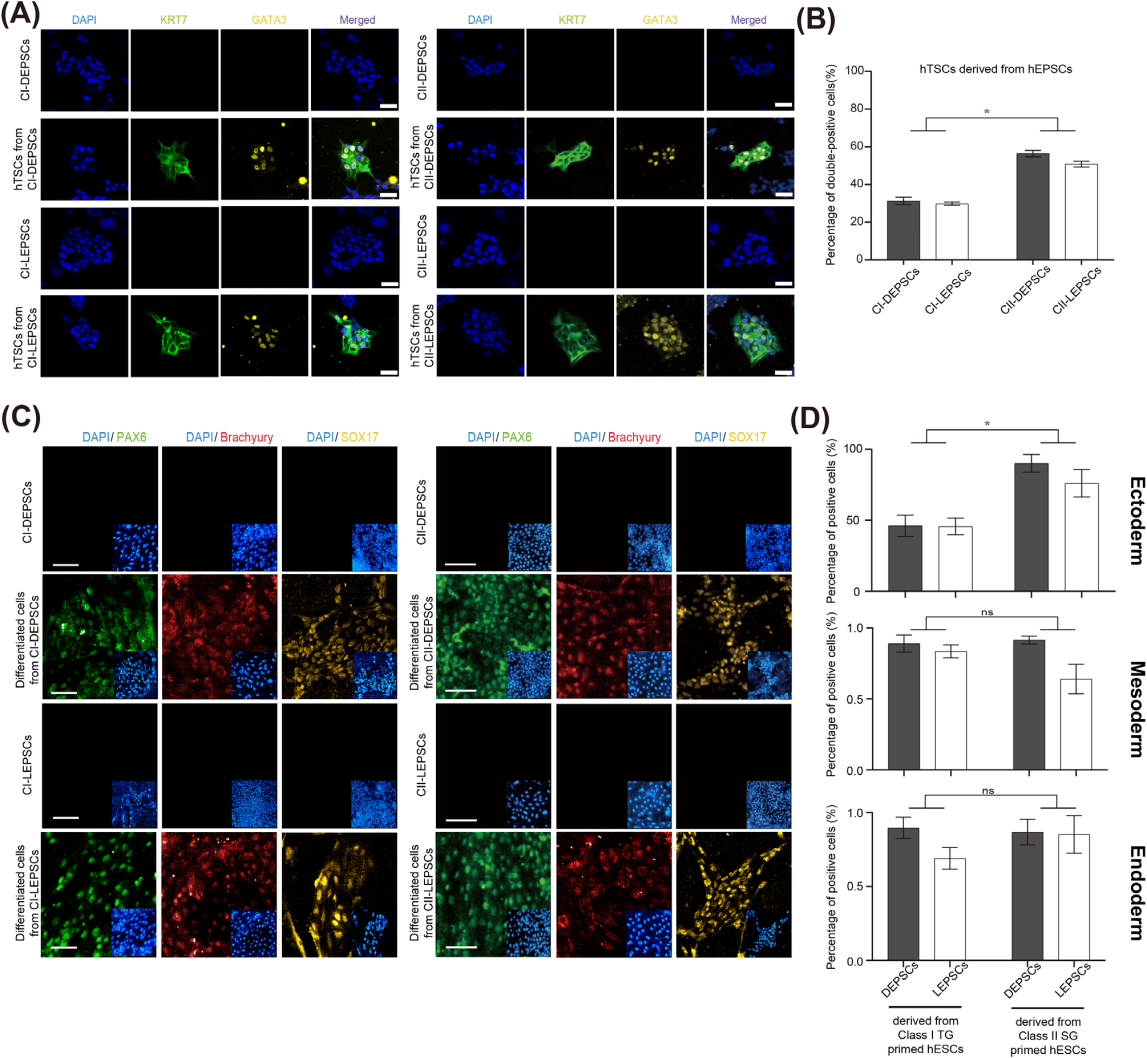
Characteristics of hTSCs and three germ layers induced from hEPSCs derived from Class I TG, Class II SG primed hESCs, respectively. (A) Representative IF staining images of hTSCs differentiated from CI‐ or CII‐hEPSCs to detect trophoblast‐specific markers. Scale Bars indicates 10 μm. (B) Quantification of percentage of hTSCs expressing both *GATA3* and *KRT7*, based on the IF staining. *p < 0.05, for comparisons of cells differentiated from CI‐DEPSCs vs. CII‐DEPSCs, CI‐LEPSCs vs. CII‐LEPSCs. (C) Representative IF staining images of ectoderm, mesoderm, endoderm lineage cells to detect specific markers. Scale bars indicate 50 μm. (D) Quantification of percentage of differentiated cells expressing lineage‐specific markers, based on IF staining. *p < 0.05, for comparisons of cells differentiated from CI‐DEPSCs vs. CII‐DEPSCs, CI‐LEPSCs vs. CII‐LEPSCs. hEPSCs, human extended pluripotent stem cells; hESCs, human embryonic stem cells; hTSCs, human trophoblast stem cells.

Considering that hEPSCs can differentiate into both the embryonic and extraembryonic lineages,^40^, ^41^ we examined the effect of the X chromosome status on differentiation. First, we differentiated CI‐ or CII‐hEPSCs into hTSCs using the established method,^49^ and cobblestone‐shaped clones appeared after 5 days (Figure S4B). Consistently, all hTSCs derived from hEPSCs highly expressed trophoblast‐specific markers, including *GATA3*, *KRT7*, *ELF5*, *TP63*, *CDX2*, and *TFAP2C* (Figures 5A and S4C), indicating a successful differentiation.^49^ To investigate whether X chromosome status influences the efficiency of hTSC derivation, we quantified the proportion of hTSCs with double expression of *GATA3* and *KRT7*, by high‐content cell analysis. We found that the percentage of *GATA3* and *KRT7* double positive cells derived from CII‐hEPSCs was significantly higher than that from CI‐hEPSCs (Figure 5B). The GSEA further validated that the hTSCs‐specific genes^51^ were expressed more readily in CII‐hEPSCs than in CI‐hEPSCs (Figure S4D). These results illustrated that CII‐hEPSCs have a better capability to differentiate into the extraembryonic lineage than CI‐hEPSCs.

Next, we differentiated hEPSCs into three embryonic germ layers using established protocols.^50^ IF staining and RT‐PCR results showed that all hEPSC‐differentiated cells correctly expressed the ectoderm (*PAX6*, *NOTCH1*, and *SOX1*), mesoderm (*Brachyury*, *MIXL1*, and *MESP1*) and endoderm markers (*SOX17*, *AFP*, and *FOXA1*) (Figure 5C and S4E). The proportions of cells expressing *PAX6*, *Brachyury*, or *SOX17* were quantified to access the differentiation efficiency of three germ layers. There is no significant difference of differentiation efficiency between DEPSCs and LEPSCs (Figure 5D). In comparison between differentiated cells from CI‐ and CII‐hEPSCs, we found that the proportion of ectoderm‐like cells from CII‐hEPSCs was significantly higher than that of CI‐hEPSCs, while they showed a similar efficiency of differentiation in mesoderm or endoderm (Figure 5D), indicating that CII‐hEPSCs had a better capability to differentiate into ectoderm than CI‐hEPSCs.

We further detected *XIST* expression in hTSCs and three germ layer cells, and observed that the differentiated cells from CI‐hEPSCs significantly downregulated *XIST* expression (Figure S4F). This downregulation may lead to failure of XCI, consistent with what we observed in the fibroblasts from hEPSCs (Figure S4A). Similar to the low *XIST* expression in CII‐hEPSCs, the differentiated cells also lowly expressed *XIST* (Figure S4F).

Collectively, these results illustrated that X chromosome status could have a decent effect on the differentiation capability of hEPSCs, and post‐XCI hEPSCs are better than pre‐XCI hEPSCs in the ability to differentiate into the embryonic or extraembryonic lineages.

## 4 DISCUSSION

There are significant differences in the X chromosome statuses and dosage compensation mechanisms between mouse and human during early development.^10^, ^52^, ^53^ Paternal imprinted XCI in early mouse embryos does not occur in human embryos.^54^, ^55^ Human pre‐implantation epiblast cells have two active X chromosomes with biallelic *XIST* expressing,^11^, ^12^ whereas *XIST* expression is absent in mouse preimplantation epiblast cells.^54^, ^55^ Therefore, a proper human model system is essential for studying the mechanism of human XCI. Previous studies showed that the silenced X chromosome in primed hESCs was reactivated after being converted to naive pluripotency. However, these naive hESCs exhibit significant heterogeneity of X chromosome status and *XIST* expression, resulting in non‐random XCI after differentiation.^35^, ^36^, ^37^, ^38^ In our previous work, we used X‐linked dual reporters to monitor X chromosome activity and established homogenous HT naive hESCs by blocking autocrine FGF signalling. HT naive hESCs have two active X chromosomes and express *XIST* bi‐allelically, resembling the X chromosome status in human pre‐implantation epiblast.^39^ These results reveal that the X chromosome status is dynamic and highly correlated with different pluripotency states.

The hEPSCs, recently established by two independent studies, have expression profiles notably different from primed or naive hESCs.^41^ They can be differentiated into embryonic or extraembryonic lineages, and can be induced to generate human blastocyst‐like structures.^43^ This expanded differentiation capability makes hEPSCs a promising model for studying early human development. However, the X chromosome status of hEPSCs has not been well‐characterized. In this study, we converted Class I TG (pre‐XCI) and Class II SG (post‐XCI) primed hESCs into hEPSCs using two culture systems.^40^, ^41^ The DEPSCs derivation method was established through the screening of a small‐molecule compound library,^40^ while the LEPSCs line was established through stepwise analysis of the pluripotency‐related signalling pathways.^41^ We observed that hEPSCs obtained through these two approaches exhibited highly similar X status and transcriptome.

XCI is a dynamic and complex event that involves various epigenetic modifications, such as DNA methylation and histone modifications. Conceivably, reactivating X chromosome requires a complete reprogramming of these epigenetic marks. The establishment of naive pluripotency is accompanied by global DNA demethylation^30^, ^33^, ^35^, ^39^, ^56^ and loss of H3K27me3 focus on previously silenced X chromosome.^39^ In this study, we found that the X chromosome status of the primed hESCs significantly impacted on that of the hEPSCs, suggesting incomplete reprogramming of X chromosome during primed to extended/expanded pluripotency conversion. On one hand, hEPSCs derived from post‐XCI primed hESCs remained post‐XCI status, with only one X chromosome being expressed, indicating that hEPSCs derivation did not reactivate the silenced X chromosome. Interestingly, in this process, the previously inactivated X chromosome lost the coating of XIST, whereas remained silenced. The underlying mechanism and epigenetic modifications need to be studied in greater details, which may provide valuable insights about human XCI. On the other hand, in hEPSCs derived from pre‐XCI primed hESCs, both X chromosomes remained active, but notable heterogeneity was observed within CI‐hEPSCs. HTG cells have two active X chromosomes with biallelic expression of *XIST* (Xa^XIST+^Xa^XIST+^), which is similar with the X chromosome status of human preimplantation epiblasts and naive hESCs.^11^, ^12^ Whereas LTG CI‐hEPSCs silenced partial X‐linked genes. Further study is necessary to dissect the mechanism of the heterogeneity, which will further improve the hEPSC culture system.

hEPSCs hold great potential for regenerative medicine due to their extended differentiation capacity and robust proliferation. However, the definition of potency of EPSCs remains controversial. A recent study suggests that mouse EPSCs are closer to the late primed pluripotent EPI, and their ability to differentiate into embryonic or extraembryonic lineages has no significant advantage compared to naive mouse ESCs.^57^ This challenges the definition of totipotency of mouse EPSCs as originally proposed. As for hEPSCs, the potency remains to be defined. At the transcriptome level, hEPSCs were more similar to primed hESCs than to HT naive hESCs (Figure S2A). Analysis of X chromosome status at the epigenetic level revealed that CII‐hEPSCs were unable to reactivate the X chromosome and maintained a highly consistent X chromosome status with post‐XCI primed hESCs. In CI‐hEPSCs derived from pre‐XCI primed hESCs, the presence of cells with partial initial XCI, known as LTG cells. These findings provide evidence that the state of hEPSCs is closer to primed pluripotency.

Our findings demonstrate that the X chromosome status of hEPSCs is affected by that of the primed hESCs from which they were derived, therefore is also quite heterogeneous. More importantly, hEPSCs with different X chromosome status have different differentiation potential. *XIST* expression was minimal in the differentiated cells derived from hEPSCs with different X status, consistent with previous findings.^22^ Our results suggest that hEPSCs with pre‐ or post‐XCI states were unable to achieve random XCI in vitro. Therefore, we should pay close attention to the epigenetic status of hEPSCs before using them to generate functional cells for therpeutic applications, considering that the function of these differentiated cells could be impaired due to abnormal XCI.

Taken together, by characterising the X chromosome status of hEPSCs, this study provides important information for the application of hEPSCs in early human development modeling and regenerative medicine.

## AUTHOR CONTRIBUTIONS

Haoyi Wang and Chenrui An conceived and designed the study. Haoyi Wang and Guihai Feng supervised the project. Ying Wang performed the experiments. Wen Sun, Chen Zhao, Xiaoxuan Hu, Shan Lu, Shiwei Cao, and Nannan Wang were involved in the methodology. Ying Wang, Ning Yang, and Chenrui An analysed the data and wrote the manuscript. Wen Sun, Xiaoxuan Hu and Shan Lu were involved in the manuscript preparation.

## FUNDING INFORMATION

National Key Research and Development Program of China, Grant/Award Numbers: 2018YFE0201102, 2019YFA0110000; Strategic Priority Research Program of the Chinese Academy of Sciences, Grant/Award Number: XDA16010503; Strategic Collaborative Research Program of the Ferring Institute of Reproductive Medicine, Ferring Pharmaceuticals, Chinese Academy of Sciences, Grant/Award Number: FIRMD181101.

## CONFLICT OF INTEREST STATEMENT

The authors declare that they have no competing interests.

## Supporting information


Figure S1. Characteristics of pluripotency of hEPSCs
A. FACS analysis showing the GFP and tdTomato activities in DEPSCs and LEPSCs (at P5 and P10) derived from Class I TG or Class II SG primed hESCs. B. Upper panel: representative RNA‐FISH and IF co‐staining images of Class I TG, Class II SG primed hESCs and their DEPSCs or LEPSCs derivatives(at P5 and P10) to detect XIST (Red), ATRX (Yellow) and H3K27me3 (Green). Scale bars indicate 10 μm; Lower panel: quantification analysis of cells having different patterns with XIST, ATRX and H3K27me3 by RNA‐FISH and IF co‐staining. C. Representative images of primed hESCs and hEPSCs (DEPSCs, LEPSCs) by AP staining. Scale bars indicate 100 μm. D. Representative images of primed hESCs and hEPSCs (DEPSCs, LEPSCs), detecting OCT4, NANOG and KLF4 protein by IF staining. Scale bars indicate 10 μm. E. Venn analysis of specific upregulated genes of H1. hEPSCs^40^, ^41^，compared with the RNA‐Seq data from HT naive hESCs^39^ and primed hESCs.^41^ F. Heatmap of hEPSC‐specific upregulated genes expression in H1 hEPSCs.^40^, ^41^ G. GO term analysis of specific hEPSCs upregulated genes. H. RT‐PCR analysis of specific hEPSCs upregulated genes expression (*SP5*, *DMD* and *WLS*) in Class I TG, Class II SG primed hESCs, CI‐hEPSCs, CII‐hEPSCs and HT naive hESCs.^39^ Data represent mean ± SD (n = 3). **P < 0.01, ****P < 0.0001, for comparisons of gene expression in primed state (Class I TG primed hESCs，Class II SG primed hESCs) vs. DEPSCs (CI, CII‐DEPSCs), LEPSCs (CI‐, CII‐LEPSCs), HT naive hESCs. I. Representative IF staining images of Class I TG, Class II SG primed hESCs and their hEPSCs derivatives to detect SP5 protein. J. FACS analysis showing the GFP and tdTomato activities in CI‐hEPSCs sorted.
Figure S2. Differential gene expression analysis of hEPSCs derived from Class I TG and Class II SG primed hESCs
A. Hierarchical clustering of gene expression in hEPSCs (DEPSCs, LEPSCs), their original WIBR3^MGT^ Class I TG, Class II SG primed hESCs and HT naive hESCs.^39^ Spearman's test was used to calculate their correlation level. B.Heatmap showing the differential genes expression of hEPSCs derived from Class I TG, Class II SG primed hESCs, respectively. C‐D. GO and KEGG analysis of upregulated genes in hEPSCs derived from Class I TG, Class II SG primed hESCs, respectively by ggplot2 in R. X‐axis represents the enrichment fold of upregulated genes. Y‐axis represents biological processes or signal pathways of upregulated genes.
Figure S3. Characteristic of specific 24 monoallelic gene expression
A. GO term analysis showing specific 24 monoallelic genes enriched with molecular functions and biological processes.
Figure S4. Characteristics of hTSCs induced from hEPSCs derived from Class I TG, Class II SG primed hESCs, respectively
A. FACS analysis showing the GFP and tdTomato activities in hEPSCs derived from Class I TG or Class II SG primed hESCs and their derivatives fibroblasts. B. Representative images of hTSCs differentiated from hEPSCs. Scale bars indicate 10 μm. C. RT‐PCR analysis of hTSCs‐specific gene expression in the differentiated cells. Data represent mean ± SD (n = 3). ****P < 0.0001. D. GSEA analysis of 839 hTSCs‐specific genes^51^ in CI‐ and CII‐hEPSCs. E. RT‐PCR analysis of ectoderm, mesoderm, endoderm lineage genes expression. Data represent the mean ± SD (n = 3). ****P < 0.0001, ***P < 0.001, **P < 0.01,*P < 0.05. F. RT‐PCR analysis of *XIST* expression in Class I TG, Class II SG primed hESCs, CI‐hEPSCs, CII‐hEPSCs and their differentiated derivatives (hTSCs and three germ layers). Data represent the mean ± SD (n = 3). ****P < 0.0001.

**Table S1.** Primers of SNPs on X chromosome
**Table S2.** Sequences of primers used for RT‐PCR

## Data Availability

The raw sequence data reported in this article have been deposited in the Genome Sequence Archive^58^ in National Genomics Data Center,^59^ China National Center for Bioinformation/Beijing Institute of Genomics, Chinese Academy of Sciences (GSA‐Human: HRA003864) that are publicly accessible at https://ngdc.cncb.ac.cn/gsa-human.
