# Supplementary material for: Characterisation of X chromosome status of human extended pluripotent stem cells
Source: Cell Prolif. 2023 May 17;56(5):e13468. doi: 10.1111/cpr.13468 (PMC10212708; doi:10.1111/cpr.13468)

Supplemental Figure 1

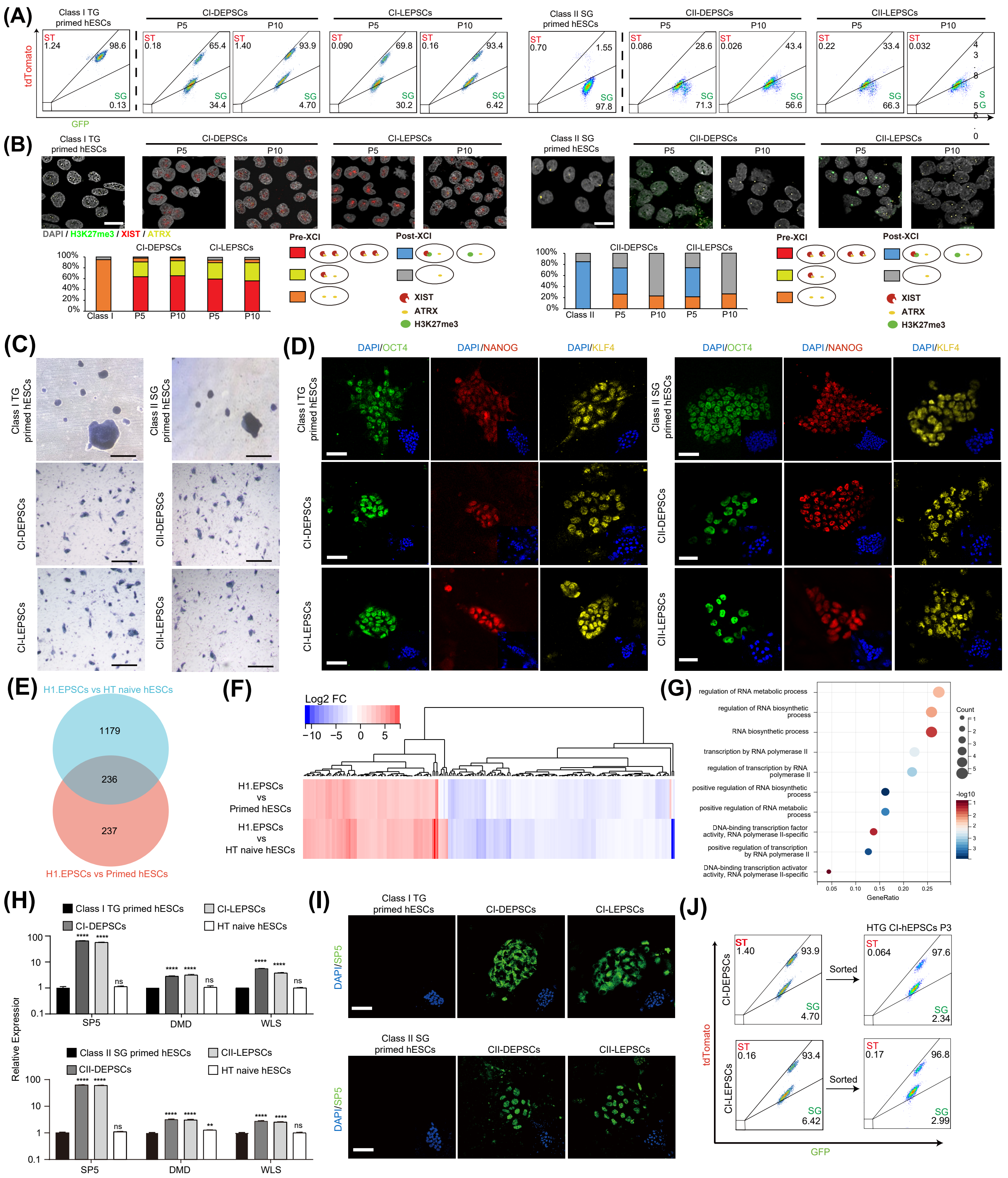

Supplemental Figure 2

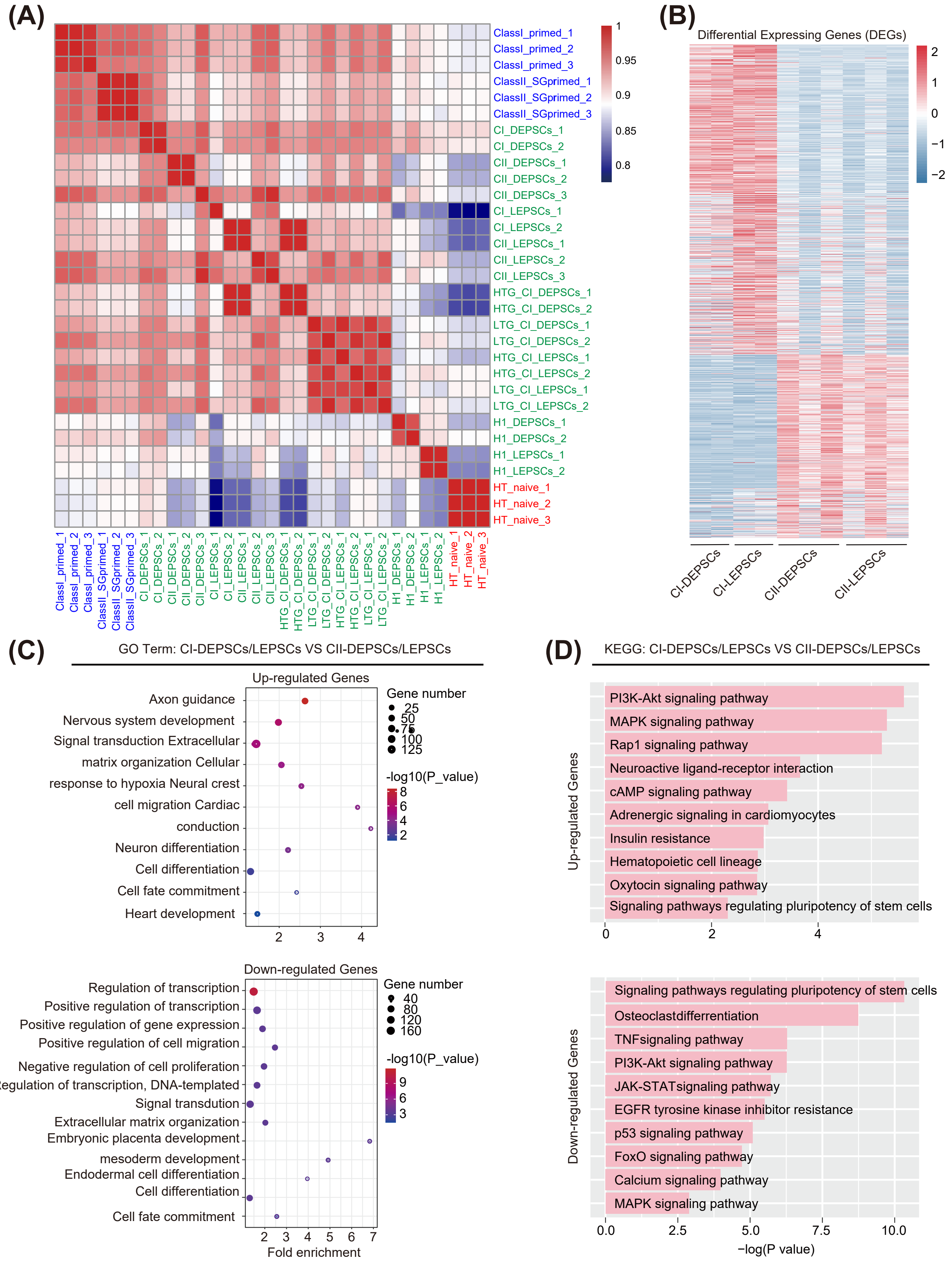

Supplemental Figure 3

(A)

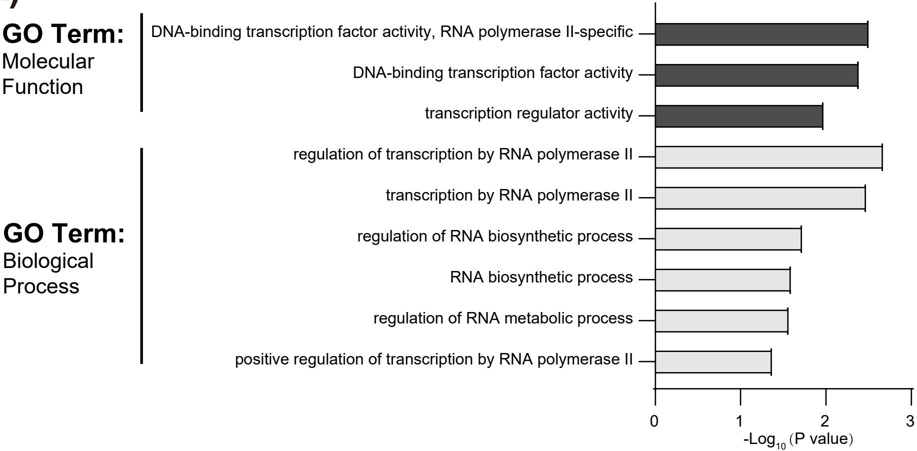

Supplemental Figure 4

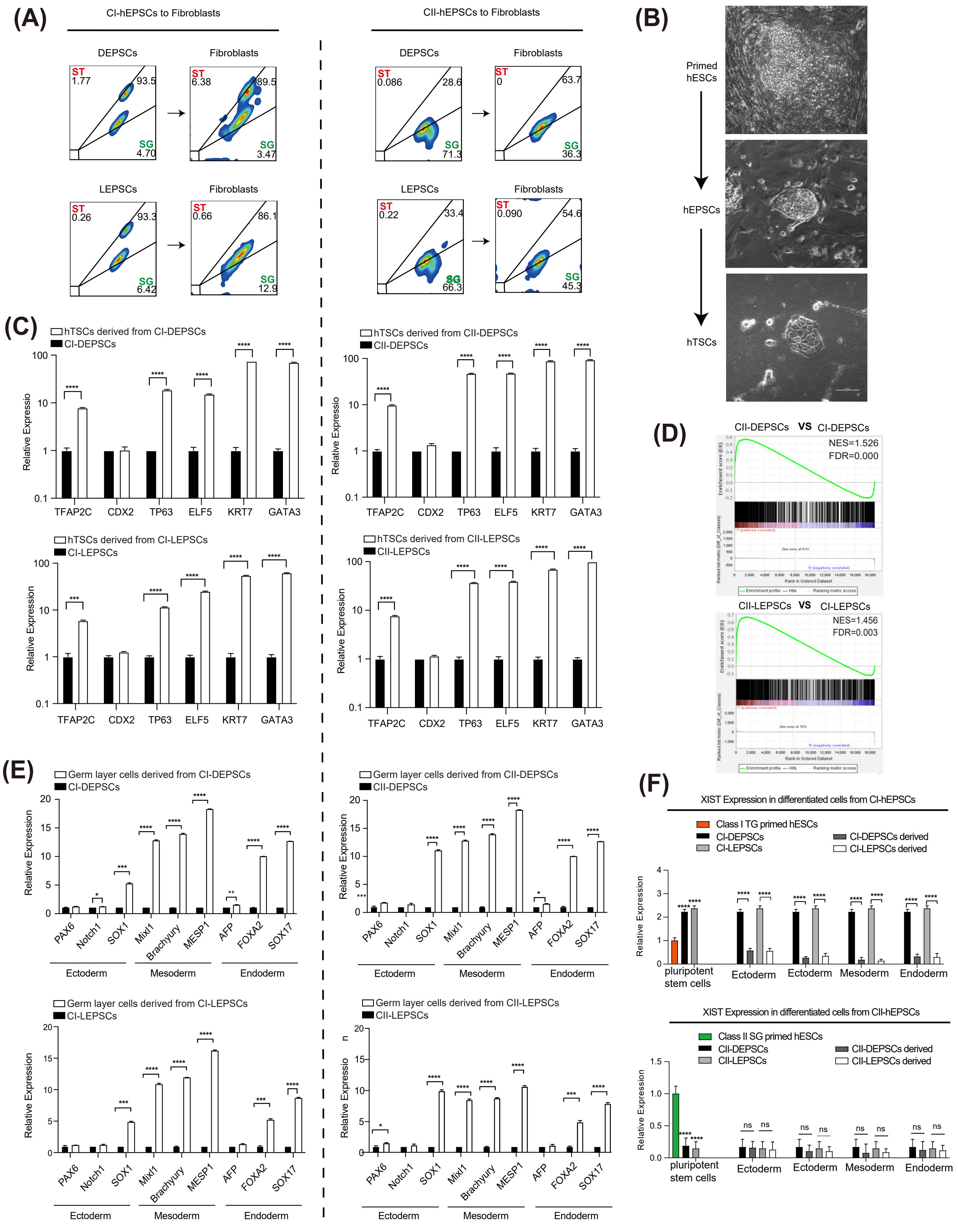

Supplement: Supplementary file 1 — Figure S1. Characteristics of pluripotency of hEPSCs A. FACS analysis showing the GFP and tdTomato activities in DEPSCs and LEPSCs (at P5 and P10) derived from Class I TG or Class II SG primed hESCs. B. Upper panel: representative RNA‐FISH and IF co‐staining images of Class I TG, Class II SG primed hESCs and their DEPSCs or LEPSCs derivatives(at P5 and P10) to detect XIST (Red), ATRX (Yellow) and H3K27me3 (Green). Scale bars indicate 10 μm; Lower panel: quantification analysis of cells having different patterns with XIST, ATRX and H3K27me3 by RNA‐FISH and IF co‐staining. C. Representative images of primed hESCs and hEPSCs (DEPSCs, LEPSCs) by AP staining. Scale bars indicate 100 μm. D. Representative images of primed hESCs and hEPSCs (DEPSCs, LEPSCs), detecting OCT4, NANOG and KLF4 protein by IF staining. Scale bars indicate 10 μm. E. Venn analysis of specific upregulated genes of H1. hEPSCs 40 , 41 ，compared with the RNA‐Seq data from HT naive hESCs 39 and primed hESCs. 41 F. Heatmap of hEPSC‐specific upregulated genes expression in H1 hEPSCs. 40 , 41 G. GO term analysis of specific hEPSCs upregulated genes. H. RT‐PCR analysis of specific hEPSCs upregulated genes expression (SP5, DMD and WLS) in Class I TG, Class II SG primed hESCs, CI‐hEPSCs, CII‐hEPSCs and HT naive hESCs. 39 Data represent mean ± SD (n = 3). **P < 0.01, ****P < 0.0001, for comparisons of gene expression in primed state (Class I TG primed hESCs，Class II SG primed hESCs) vs. DEPSCs (CI, CII‐DEPSCs), LEPSCs (CI‐, CII‐LEPSCs), HT naive hESCs. I. Representative IF staining images of Class I TG, Class II SG primed hESCs and their hEPSCs derivatives to detect SP5 protein. J. FACS analysis showing the GFP and tdTomato activities in CI‐hEPSCs sorted. Figure S2. Differential gene expression analysis of hEPSCs derived from Class I TG and Class II SG primed hESCs A. Hierarchical clustering of gene expression in hEPSCs (DEPSCs, LEPSCs), their original WIBR3MGT Class I TG, Class II SG primed hESCs and HT naive [file CPR-56-e13468-s001.pdf]
