# Supplementary material for: Characterisation of X chromosome status of human extended pluripotent stem cells
Source: Cell Prolif. 2023 May 17;56(5):e13468. doi: 10.1111/cpr.13468 (PMC10212708; doi:10.1111/cpr.13468)
Supplement: Supplementary file 2 — Table S1. Primers of SNPs on X chromosome Table S2. Sequences of primers used for RT‐PCR [file CPR-56-e13468-s002.docx]

**Table S1. Primers of SNPs on X chromosome**

| Gene | Sequences of Primer（5’-3’） |
| --- | --- |
| *MBTPS2* | F:CAGCATGTGAGCAGAGGGAG |
| *MBTPS2* | R:ATAGCTTCACACTGGCCTGC |
| *BRWD3* | F:AGAAACCTGAGTGCTACCCA |
| *BRWD3* | R:TGTATGTTTGTGGTAATGGGGA |
| *SLCA8* | F:GAGCTCAGGACGCAGATGTC |
| *SLCA8* | R:CTCCCTCCTTCCCTTCCTCA |

**Table S2.** **Sequences of** **primers used for RT-PCR**

| Gene | Sequences of Primer（5’-3’） |
| --- | --- |
| *GAPDH* | F:GAGTCAACGGATTTGGTCGT |
| *GAPDH* | R:TTCCCGTTCTCAGCCTTG |
| *KRT7* | F:AGGATGTGGATGCTGCCTAC |
| *KRT7* | R:CACCACAGATGTGTCGGAGA |
| *GATA3* | F:TGCAGGAGCAGTATCATGAAGCCT |
| *GATA3* | R:GCATCAAACAACTGTGGCCAGTGA |
| *FOXA2* | F:GGCCCAGTCACGAACAAAGC |
| *FOXA2* | R:CCCAAAGTCTCCACTCAGCCTC |
| *AFP* | F:CCCGAACTTTCCAAGCCATA |
| *AFP* | R:TACATGGGCCACATCCAGG |
| *SOX17* | F:AAGAAACCCTAAACACAAACAGCG |
| *SOX17* | R:TTTGTGGGAAGTGGGATCAAGAC |
| *MESP1* | F:TGTACGCAGAAACAGCATCC |
| *MESP1* | R:TTGTCCCCTCCACTCTTCAG |
| *Brachyury* | F:GCTTCAAGGAGCTAACTAACGAG |
| *Brachyury* | R:CCAGCAAGAAAGAGTACATGGC |
| *Mixl1* | F:GGAGCTCGTCTTCCGACAGA |
| *Mixl1* | R:TTGAGGATAAGGGCTGAAATGAC |
| *SOX1* | F:ATACCCCCAAAATGCATCAA |
| *SOX1* | R:GGAAACGGGCTTTTCTCTCT |
| *PAX6* | F:GTTCCCTGTCCTGTGGACTC |
| *PAX6* | R:ACCGCCCTTGGTTAAAGTCT |
| *Notch1* | F:TGCCAGACCAACATCAAC |
| *Notch1* | R:CTCATAGTCCTCGGATTGC |
| *TP63* | F:AGAAACGAAGATCCCCAGATGA |
| *TP63* | R:CTGTTGCTGTTGCCTGTACGTT |
| *ELF5* | F:TAGGGAACAAGGAATTTTTCGGG |
| *ELF5* | R:GTACACTAACCTTCGGTCAACC |
| *TFAP2C* | F:CTGTTGCTGCACGATCAGACA |
| *TFAP2C* | R:CTCAGTGGGGTTCATTACGGC |
| *CDX2* | F:AGCCCCGCAGACTACCAT |
| *CDX2* | R:TCTTTCGTCCTGGTTTTCACT |

| Gene | Sequences of primers（5’-3’） | |
| --- | --- | --- |
| *XIST* | F:ATGCCTGGCACTCTAGCACT | |
| *XIST* | R:GCAAGAGAAACATGGAAATGG |  |
| *WLS* | F:GTTGGCTCCTTCTGCCTCTTCA  GTTGGCTCCTTCTGCCTCTTCA  GTTGGCTCCTTCTGCCTCTTCA  GTTGGCTCCTTCTGCCTCTTCA  GTTGGCTCCTTCTGCCTCTTCA  GTTGGCTCCTTCTGCCTCTTCA |  |
| *WLS* | R:AGGCAGATTCCAGCCACGATGA |  |
| *DMD* | F:GCTCAACCATCGATTTGCAGCC |  |
| *DMD* | R:TTCAGCCTCCAGTGGTTCAAGC |  |
| *SP5* | F:GAAGAAGCAGCACGTGCCAC |  |
| *SP5* | R:CAGAAGAGCCAGTTGCACACGA |  |
